# Heavy metals in carabids (Coleoptera, Carabidae)

**DOI:** 10.3897/zookeys.100.1529

**Published:** 2011-05-20

**Authors:** Ruslan O. Butovsky

**Affiliations:** Tula State Pedagogical University, 300026, Lenina prospekt, 125, Tula, Russian Federation

**Keywords:** Heavy metals, carabid beetles, pollution, accumulation

## Abstract

Carabid beetles (Coleoptera, Carabidae) are one of the most studied soil groups in relation to heavy metal (HM) accumulation and use for bioindication of environmental pollution. Accumulation of Zn and Cu in carabid beetles was species-, sex- and trophic group-specific. No differences were found in HM contents between omnivorous and carnivorous species. The use of carabid beetles as indicators of HM accumulation appears to be rather limited.

## Introduction

Because of the increasing impacts of chemicals on terrestrial and soil ecosystems, assessment of environmental quality by bioindicators is of particular interest.

Carabid beetles are traditionally used as bioindicators of anthropogenic stresses for a number of reasons. They inhabit most terrestrial ecosystems. The ecology and systematics of the group are well studied. Sampling methods are simple and universal. And the data collected by different researchers are comparable.

In soil trophic webs, carabid beetles play an extremely important role as non-specialized predators and 2nd order consumers.

There are numerous publications demonstrating structural changes in carabid communities due to different anthropogenic impacts caused by motorways, metallurgic smelters, and recreation (see review by Butovsky 2001). In addition, several studies on heavy metal accumulation in carabid beetles have been performed in different ecosystems (see review by Butovsky 1997).

This paper reviews the literature data on heavy metal (HM) impacts on carabid beetle communities and also considers the use of carabids as indicators of HM accumulation.

## Results and discussion

### HM contents limits

The mean HM content in carabids decreased in the following sequence Fe > Zn > Cu > Mn > Pb > Cd (Butovsky 1997). The most toxic elements (Pb and Cd) were not accumulated in large quantities, in contrast to the accumulated Zn and Cu (Andrews and Cooke 1984; Beyer et al. 1985; van Straalen and van Wensem 1986; Roth 1993; Jelaska et al. 2007).

The concentration ratios of Cd : Pb : Mn : Cu : Zn : Fe in an average carabid beetle were 1 : 2.5 : 7 : 17 : 29.5 : 93. In other words, an average carabid beetle may contain 93 times more Fe than Cd and 37 times more Fe than Pb (Butovsky 1997).

### HM contents in different genera

Variation coefficients of HM in carabids were variable and dependent on the carabid genus and type of HM (Table 1). In *Carabus* spp. for Pb, Zn and Cu the coefficient was 17.9% (5.2–32.5%) and in *Pterostichus* spp. 29.9% (6.3–48.2%). (Table 1).

The analysis of 14 HMs in 28 carabid species revealed that the variability of HM in *Carabus* spp. was 26% and in *Pterostichus* spp. 42% (Stepanov et al. 1987). The authors aggregated all predacious carabids for bioindication as a uniform group, thus ignoring differences at species level.

**Table 1.** Inter-generic variability in HM contents (ppm) in carabid beetles (by different authors after Butovsky 1997).

*Genera*	*N*	*Heavy metals, ppm*					
		*Cd*	*Cu*	*Fe*	*Mn*	*Pb*	*Zn*
*Abax*	3	0.1*	15.9	N/A	N/A	3.1+1.7	62.8
*Agonum*	2	0	25.8+ 7.9	532.3	24.3	4.9+4.9	95.0+14.4
*Calathus*	4	1.0+0.3	57.2+30.8	58.7+ 49.0	29.1+9.3	6.7+1.9	89.2+21.1
*Carabus*	9	0.1+0.1	16.9+ 0.9	333.9	N/A	4.3+1.4	96.1+15.3
*Harpalus*	1	0	23.3	N/A	N/A	0	130
*Leistus*	2	2.9+1.1	30.1	N/A	N/A	7.8	118.6
*Loricera*	1	N/A	N/A	N/A	N/A	1.9	N/A
*Notiophilus*	2	1.7+0.3	27.5+ 1.3	117.1+109.9	29.2+2.8	1.7+0.3	77.9+10.5
*Poecilus*	2	0.1+0.1	16.3	N/A	N/A	4.2+4.2	118.7+30.7
*Pseudo-ophonus*	1	0	17.3+ 2.1	461.2	N/A	3.0+3.0	92.3+ 6.4
*Pterostichus*	5	0	29.5+10.4	436.3+ 37.3	N/A	2.9+1.4	116.2+ 7.4

**N** – number of species;

* - one replicate;

**N/A** - not available.

### HM contents in small, medium and large carabid species

A positive correlation between body mass and Pb content was found in beetles of the genus *Carabus* (Emets and Zhulidov 1983) and three other species: *Calathus melanocephalus*, *Notiophilus biguttatus*, *Notiophilus rufipes* (but not for Zn or Cd) (van Straalen and van Wensem 1986).

I subdivided the collected carabids into three groups: (1) with body mass (B) less than 15 mg (genera *Agonum*, *Leistus*, *Loricera*, *Notiophilus*, *Calathus*); (2) with 15<B<50 mg (genera *Pseudophonus*, *Poecilus*, *Pterostichus*, *Abax*, *Harpalus*) and (3) with B>50 mg (genus *Carabus*) (Table 2).

Mean HM content (at least for Zn, Pb and Mn) was not dependent on the mass/size of carabid beetles. Medium-sized species contained more Fe compared to small-sized species and small species contained more Cd and Cu compared to medium-sized and large species.

**Table 2.** Heavy metal content (*ppm*) and dry body mass of carabid beetles (Butovsky 1997).

*Heavy metals*	**Body mass,** mg		
	*<15*	*15–50*	*>50*
Cd	1.4+0.6	0.5+0.3	0.1+0.1
Cu	35.1+7.4	20.5+2.6	16.9+0.9
Fe	236.0+149.1	448.7+12.4	333.9
Mn	26.7+2.4	29.1	-
Pb	4.6+1.2	2.6+0.7	4.3+1.4
Zn	95.2+8.6	104.0+11.9	96.1+15.3

### HM contents in males and females

In most studies no clear pattern of HM contents in males or females of carabid species were observed (Roth 1993; Butovsky 1995, 2001).

In some species, males contained more metals (Pb, Zn, Cd, Cu, Mn, Fe, Co, Ni, Sr, Cr, Al) than females (Stepanov et al. 1987). In another study on ten species, males accumulated more Cu than females, which, in contrast, accumulated more Cd than males (Purchart and Kula 2007).

Sex-specific differences were found in six carabid species (*Poecilus cupreus*, *Pterostichus melanarius*, *Pterostichus niger*, *Pseudophonus rufipes*, *Carabus nemoralis* and *Carabus granulatus*), while females contained more Zn than males (Butovsky 1994).

Microelement (Na, Mg, K, Ca) concentrations were higher in females compared to males in populations of *Agonum dorsale* and *Agonum sexpunctatum* (Novak 1989).

### HM in different species

No regular pattern was found in studies of HM contents in dozens of carabid species published by numerous authors (reviewed by Butovsky 2001). The contents were variable and species-specific.

### HM and feeding behavior

Omnivorous species (*Harpalus* spp., *Amara* spp.) contained more Cu but less Zn than carnivorous species (*Pterostichus* spp., *Carabus* spp.) in roadside ecosystems (Butovsky 1995).

The effects of feeding ecologies were evident only for the essential elements: carnivores (*Calathus* spp., *Pterostichus cupreus*, *Pterostichus melanarius*) had significantly higher contents of Zn, Cu and Mn than omnivores (*Harpalus* spp., *Pterostichus rufipes*). No differences were found for Pb and Cd (Purchart and Kula 2007).

### HM and seasonal changes

Seasonal differences in abundance, species composition, and age structure of invertebrates may lead to high variability in HM contents in carabid beetles, and the highest variability can be expected at highly polluted sites (Hunter et al. 1987).

Seasonal changes in Cd contents were not found for *Notiophilus biguttatus*, but higher concentrations were observed in *Calathus melanocephalus* in autumn, likely due to a peak of reproductive activity (Janssen 1991).

I found a decrease of Zn and Cu contents in the dominant species *Pterostichus cupreus* and *Pterostichus melanarius* in roadside ecosystems of the Moscow region at the end of the season (Butovsky 1994, 1995).

In roadside populations of *Pterostichus oblongopunctatus* the Zn contents of over-wintered beetles was higher than in newly hatched ones (Emetz and Kulmatov 1983).

In ten carabid species, Zn and Cu contents during the spring were higher than in autumn. The authors speculated that in the period of increased feeding activity (spring), the elements were stored in body fat, while during sexual activity and wintering they were mobilized and excreted. The composition of a population with regards to the fraction of juvenile specimens, active feeders, or reproducing individuals may have a considerable effect on the seasonal dynamics of the metals (Purchart and Kula 2007).

### Bioaccumulation and biomagnification

Carabids are relatively poor accumulators of heavy metals, particularly the most toxic ones, such as cadmium or lead (Butovsky 1997). Among carabids the most active HM accumulators were represented by *Carabus* spp. (concentration factor, *Cf* = 5.2–6.7) (Emetz and Zhulidov 1983). Less accumulation was observed in omnivorous species (*Cf* = 1.1–2.0) (Butovsky 1995).

In contrast, the highest concentrations of non-essential metals (Cd and Pb) were found in carnivorous carabid beetles together with earthworms and oribatid mites in the vicinity of a metallurgic smelter (van Straalen et al. 2001).

In putative trophic chains, carabids as non-specialized predators accumulated less copper and zinc (*Cf* = 0.54 and 0.21 respectively) than specialized predators (like *Coccinellidae*, *Syrphidae*) (0.67 and 0.99 respectively) and specialized parasitoids (*Alloxystidae*, *Pteromalidae*) (1.07 and 2.08 respectively). These differences probably reflected the increase of trophic adaptation to elevated concentrations of HM in non-specialized predators, specialized predators and parasitoids (Butovsky and van Straalen 1995).

### Mechanisms of HM detoxification

As in other holometabolic insects, carabid beetles possess various detoxification systems, which can segregate metals and turn it into inactive forms (Hopkin 1989) although there has not been much research conducted on these systems in beetles. One may expect that HM are stored in metal-containing granules in the hindgut wall and can be excreted with the faeces.

Compared to other groups of soil invertebrates, carabid beetles are characterized by low accumulation and high excretion rates of cadmium (Table 3).

The concentrations of Pb differed between the exoskeleton and the soft tissues in the carabid body. Up to 63–82% of Pb was accumulated in the exoskeleton (Roberts and Johnson 1978). Additional data confirm that HM (in particular, Cd) were accumulated in the exoskeleton and lost during larval molts (Lindquist et al. 1995). That may explain the fact that in many studies, carabid larvae contained more metals than imagos (Carter 1983).

Females from contaminated sites have elevated activities of some enzymes (glutathione-S-transferase and carboxyl-esterase), but males do not (Stone et al. 2002).

The fat concentration in carabids collected from polluted sites was lower when compared to reference sites. Presumably, HM excretion requires energy, thus restricting the accumulation of fat (Lindqvist and Block, 2001).

Adaptation (in terms of HM accumulation and excretion) did not occur in carabids inhabiting chronically polluted sites and obviously had no genetic basis (Lagisz and Laskowski 2007).

**Table 3.** Cd “accumulators” and “disseminators” in soil invertebrate communities (van Straalen and van Wensem 1986; van Capelleveen 1987; Janssen 1991; Butovsky et al. 1999)

*High Cd contents*	*Low Cd contents*
Isopoda	Orabitida (fam. Notaspididae)
Opiliones	Lithobiidae
Lyniphiidae	Collembola (Entomobriidae)
Pseudoscorpions	Carabidae
Gamasidea	Staphylinidae
Oribatida (fam. Camisiidae)	Gryllidae
Geophylidae	Tettigoniidae
Diplopoda	
Collembola (fam. Onychiroidea)	

## Conclusion

Carabid beetles constitute one of the most appropriate invertebrate groups for the study of “ecological” effects of different anthropogenic stressors of soil communities, and the changes in carabid community dominance, diversity, abundance, sex ratio etc. have been used as bioindicators in numerous studies (Butovsky 2001).

On the other hand, carabids are relatively poor HM accumulators (being both holometabolic insects and predators). They may contain elevated amounts of HM in polluted sites compared to referent sites, but results are variable and no accurate assessments of contamination levels can be made.

Our extensive research in roadside ecosystems showed that HM contents in carabids did not correlate with their relative abundance or distance from the motorway (Butovsky 1995) or a metallurgic smelter (van Straalen et al. 2001).

More research is obviously needed on HM stress on carabids, e.g. detoxification, genetic resistance, physiology and demography.
